# Water Influence on the Physico-Chemical Properties and 3D Printability of Choline Acrylate—Bacterial Cellulose Inks

**DOI:** 10.3390/polym15092156

**Published:** 2023-04-30

**Authors:** Veronika S. Fedotova, Maria P. Sokolova, Vitaly K. Vorobiov, Eugene V. Sivtsov, Natalia V. Lukasheva, Michael A. Smirnov

**Affiliations:** 1 Institute of Macromolecular Compounds, Russian Academy of Sciences, V.O. Bolshoi Pr. 31, 199004 St. Petersburg, Russiavrbvrbvrb@mail.ru (V.K.V.); 2 Saint Petersburg State Institute of Technology, Moskovsky Prospekt 24-26/49, 190013 St. Petersburg, Russia

**Keywords:** bacterial cellulose nanofibers, polymerizable ionic liquids, water, cellulose structure, molecular dynamics simulations, rheological properties, 3D printing

## Abstract

The aim of this work was to study the influence of water as a co-solvent on the interaction between a polymerizable ionic liquid—choline acrylate (ChA)—and bacterial cellulose. Bacterial cellulose dispersed in ChA is a new type of UV-curable biopolymer-based ink that is a prospective material for the 3D printing of green composite ion-gels. Higher cellulose content in inks is beneficial for the ecological and mechanical properties of materials, and leads to increased viscosity and the yield stress of such systems and hampers printability. It was found that the addition of water results in (1) a decrease in the solvent viscosity and yield stress; and (2) a decrease in the stability of dispersion toward phase separation under stress. In this work, an optimal composition in the range of 30–40 wt% water content demonstrating 97–160 Pa of yield stress was found that ensures the printability and stability of inks. The rheological properties of inks and mechanical characteristics (0.7–0.8 MPa strength and 1.1–1.2 MPa Young’s modulus) were obtained. The mechanism of influence of the ratio ChA/water on the properties of ink was revealed with atomic force microscopy, wide-angle X-ray diffraction studies of bacterial cellulose after regeneration from solvent, and computer simulation of ChA/water mixtures and their interaction with the cellulose surface.

## 1. Introduction

Additive manufacturing (3D printing) is a very promising technique that allows the layer-by-layer production of metal [1], ceramic [2] or polymer [3] products with arbitrary geometry and a spatial distribution of properties [4,5,6]. Modern studies in this field are often devoted to the elaboration of compositions for the printing of smart and bio materials [7,8], using greener chemicals [9], especially polymers and gels [10,11], that are prospective for tissue engineering [12], soft-robotics [13] and new generations of electrochemical devices [14]. In this context, the replacement of synthetic polymers with natural ones (cellulose [15,16] or other polysaccharides) is clearly one of the major directions [4]. The important task is the turning of material composition and biopolymer processing techniques in order to attain optimal stability and rheological properties of inks before printing [17], as well as the mechanical and functional properties of printed objects [18]. For the designation of compositions applicable for extrusion 3D printing the term “ink” is used as this is similar in principle to common inkjet printing that employs liquid inks. Owing to the high viscosity of the composition, the filament coming out of the nozzle retains its shape and three-dimensional products could be made, while inkjet printers produce only 2D objects.

Cellulose cannot be processed via melt [19,20], while cellulose nanofibers (CNF)- or nanocrystals (CNC)-based hydrogels demonstrate suitable yield stress, shear thinning behavior and fast viscosity recovery (thixotropy) after extrusion, making them applicable for 3D printing at room temperature [21]. The high amount of surface hydroxyl groups and entanglements are responsible for the rheological properties of cellulose-based hydrogel [22]. It was demonstrated that in the case of dispersion containing 2 wt% of CNF at an increasing shear speed from 10^−4^ to 10^4^ s^−1^, the viscosity of hydrogel decreases from 10^6^ Pa·s by six orders of magnitude, which is enough for printing [23]. At the same time, the fixation of the shape of printed gels needs post processing, such as by drying at ambient conditions [17] or immersing the printed object in CaCl_2_ (crosslinker) with cetyltrimethylammonium bromide with subsequent drying on a Teflon film at room temperature [23]. Both of these processes can result in the changing of the product geometry. Additionally, the use of gels with a CNF content higher than 2 wt% was not reported. This is connected with increased yield stress due to cooperative interaction via hydrogen bonding between CNF and the extremely high pressure needed for the extrusion of ink [24]. On the other hand, for the strengthening of hydrogels the increase in CNF content in inks is beneficial. This can be done by replacing CNF with CNC, for which the printing of hydrogel with 20 wt% of cellulose was reported [25]. This is connected with a significantly lower aspect ratio of CNC in comparison to CNF, and thus less entanglement. However, CNC forms reinforce the network more weakly in the resulting hydrogel. Thus, investigations of ways to increase the CNF content in 3D printable inks are significant for the elaboration of printed hydrogels with increased mechanical characteristics.

A high viscosity of CNF hydrogels is connected with moderate activity of water in the formation of hydrogen bonds with cellulose, compared to cellulose–cellulose interactions. Ionic liquids (ILs) are more active in the formation of hydrogen bonds with cellulose [26,27], which results in the better stabilization of dispersions or even the dissolution of cellulose, which allows higher concentrations of cellulose in inks to be achieved compared to water-based systems. For example, an ink containing 4 wt% of cellulose in 1-ethyl-3-methylimidazolium acetate ([C_2_mim]Ac) was successfully used for 3D printing [28]. However, in this case another problem arises: the high intrinsic viscosity of ILs leads to increased yield stress and a viscosity of inks that hampers the direct application of IL-cellulose inks in 3D printing [29]. This problem can be solved by the addition of a co-solvent that can significantly reduce the viscosity of ILs [30]. The most often studied co-solvents for this purpose are dimethyl sulphoxide (DMSO) [31] and water [32]. At the same time, both of them are non-solvents for cellulose and reduce the interaction between ILs and cellulose. DMSO was applied as a co-solvent for [C_2_mim]Ac, and resulted in decreasing the viscosity of cellulose-based inks and making them more suitable for 3D printing [33]. The same results on the viscosity of IL and cellulose solution in IL with the addition of DMSO were also reported in [34,35]. At the same time, the addition of water did not demonstrate so obvious an effect on the rheology of IL-cellulose inks. On the molecular level, water molecules can form a hydrate shell on IL via ion–dipole interactions, thus hampering hydrogen bond formation between cellulose and IL, decreasing its ability to stabilize or dissolve cellulose. As a result, the agglomeration of cellulose occurs, and the viscosity and yield stress of ink increase [36]. Experimentally, this effect was demonstrated in reference [37] for a cellulose/water/[C_2_mim]Ac system. It was found that cellulose in a binary solvent was close to a suspension, not a solution [37]. The molecular dynamics (MD) simulations provided information about the intermolecular interactions between cellulose and IL. It was confirmed that the introduction of water changed the structural organization of [C_2_mim]Ac and disrupted the interactions between IL and cellulose [38]. In another work [39], a simulation showed that the hydrogen bonds between the anion of IL (chloride) and the hydroxyl groups of cellulose disrupt due to the hydration of chloride ions or become stronger in the presence of DMSO.

All the mentioned works concerning the use of co-solvents were conducted with imidazolium-based ILs. However, the degrees of crystallinity and polymerization of cellulose decreases when this type of solvent is used [40,41,42]. This is not favorable for the mechanical properties of materials after printing. At the same time, choline-based IL can form dispersions of CNF without a significant influence on their crystalline structure, fibrillar morphology and degree of polymerization [40,43], making choline-based ILs attractive for ink preparation. The successful application of polymerizable choline acrylate (ChA) as a base for cellulose-containing inks for 3D printing was demonstrated earlier [40]. Additional benefits of this system are the easy shape fixation via UV-induced polymerization and high shape fidelity because of the low volatility of IL. Taking into account all above considerations, it was hypothesized that in the case of a cellulose-ChA system with high cellulose content and unsuitable (excessive) yield stress, the amount of water should be enough to make the yield stress suitable for 3D printing, but not enough for significant cellulose aggregation and the loss of system homogeneity. Because this idea can also be applied beyond ChA systems, the study of molecular mechanism of interactions between ink components is of prime importance. Additionally, simulations of the interaction in choline-based ILs/cellulose dispersions in the presence of water in tight connections with experimental study have not been reported previously. Thus, the aim of this work was to check the hypothesis by studying the 3D printability and rheology of ChA/water/CNF dispersions with different water contents, as well as the structure of CNF generated by them (atomic force microscopy and wide-angle X-ray diffraction studies were used). In combination with the estimation of molecular mechanisms that determine the physico-chemical behavior of materials under study, MD simulations of ChA/water mixtures and their interaction with cellulose surface were performed and analyzed.

## 2. Materials and Methods

### 2.1. Materials

For the synthesis of choline acrylate, choline chloride (ChCl, CAS 67-48-1, purity > 99%) was purchased from Glentham Life Sciences Ltd. and vacuum-dried at 60 °C before use. Acrylic acid (CAS 607-061-00-8, purity > 99%) was purchased from Sigma-Aldrich (Prague, Czech Republic). KOH (purity > 99%) and methanol (purity > 99.5%) were bought from VEKTON (Saint Petersburg, Russia); NaOH (CAS 1310-73-2) was bought from NevaReaktiv (Saint Petersburg, Russia). To obtain bacterial cellulose, a lyophilized culture of *Acetobacter xylinum* was used, which was purchased from the All-Russian collection of industrial microorganisms (National Bioresource Center, GosNIIgenetika, Moscow, Russia). Peptone and D-mannitol (CAS 69-65-8) obtained from LenReaktiv (Saint Petersburg, Russia) and yeast extract from the Research Center for Pharmacotherapy (Saint Petersburg, Russia) were used to prepare the culture medium.

### 2.2. Methods

#### 2.2.1. Synthesis of Choline Acrylate

Choline acrylate (ChA) was obtained according to the procedure described in reference [40], which included two stages. Succinctly, the synthesis consisted of two ion exchange reactions, which were carried out in a thermostatically controlled vessel at 0 °C. At the first stage, choline hydroxide (ChOH) was obtained by mixing 15.245 g of ChCl and 30 wt% KOH solution in methanol. In the second stage, the ChOH solution was mixed with 8.656 g of acrylic acid (AA) (taken in a small excess). The residual ChCl content in ChA was evaluated using the gravimetric method by carefully collecting, drying and weighing the KCl obtained at the first stage. It was found that the yield of KCl was 98 wt%. Thus, the content of unreacted ChCl can be estimated as 1.6 wt%. Further, methanol was distilled from the reaction mixture using a rotary evaporator at 40 °C and reduced pressure. After the removal of methanol, the temperature was raised to 70 °C to remove excess AA. As a result, a white gel-like liquid was obtained.

The chemical structure of the synthesized ChA was confirmed by ^1^H and ^13^C NMR spectroscopy using the NMR Fourier spectrometer AVANCE II-500WB (Bruker, Billerica, MA, USA). D_2_O was used as a solvent. The spectra were the same as those reported earlier in [40].

#### 2.2.2. Measurement of Solvent Density

Data on the density of pure ChA and its aqueous solutions (concentration of water in ChA/water mixture was 0, 10, 20, 30, 40 or 50 wt%) were obtained in the temperature range of 298–328 K using glass pycnometers. The mass of the liquid was determined using an AP225WD (Shimadzu, Kyoto, Japan) microbalance with an accuracy of 0.01 mg. Temperature control after filling the pycnometer was carried out using the LT-400 precise thermostat (LOIP, St. Petersburg, Russia) with an accuracy of ±0.01 K. Further in the text, the ChA/water mixture was designated as S-n (n indicates the water content in the mixture and is equal to 0, 10, 20, 30, 40 or 50 wt%).

#### 2.2.3. Preparation of Dispersions and Regeneration of Cellulose

Bacterial cellulose (BC) was produced from a culture of Acetobacter xylinum, as described in our previous work [43]. Briefly, the culture was statically incubated at 27 °C in a 1 L aqueous medium containing 25 g of D-mannitol, 5 g of yeast extract and 3 g of peptone until a BC pellicle with a thickness of about 1 cm was formed. After that, the pellicle was purified with a 0.5 wt% NaOH aqueous solution (100 °C) and rinsed with distilled water until a neutral pH was achieved. Next, the BC pellicle was mechanically disintegrated with a blender and then lyophilized.

The BC dispersions were prepared by mixing BC with ChA. Then, water was added to BC/ChA in order to achieve concentrations of 20, 30, 40 and 50 wt% in the ChA/water mixture. The resulting BC content in all the dispersions was chosen as 4 wt% as the maximum that can be dispersed in ChA. The obtained viscous masses were stirred for 30 min at 60 °C until the cellulose was completely wetted with a solvent. The dispersions were homogenized by repeatedly passing them through a nozzle with an outlet diameter of 0.58 mm. Further in the text, the systems are designated as CNF-n (n indicates the water content in the solvent and is equal to 0, 20, 30, 40 and 50 wt%). The cellulose was regenerated from the mixed solvent by diluting the dispersion with excess water and separated using a centrifuge (Digisystem Laboratory Instruments Inc., New Taipei, Taiwan) operating at 4000 rpm for 20 min. Removal of the residual solvent was achieved by repeated washing of the cellulose with distilled water and centrifugation. Then, the cellulose was dried on a slide glass and used for further investigation of its structure.

#### 2.2.4. Rheological Studies

Rheological measurements of both anhydrous and water-containing BC/ChA dispersions were performed using a Physica MCR 302 rheometer (Anton Paar, Graz, Austria) in the plane–plane measuring system with a diameter of 25 mm and a gap of 0.4 mm. Data processing was performed in the program RHEOPLUS/32 V.3.62 (Anton Paar, Ostfildern, Germany). Steady shear measurements of CNF-0 and water-containing BC/ChA dispersions were carried out at 25 °C with an increase in the shear rate.

The dynamic rheological behavior was studied at 25 °C. The limit of the linear viscoelasticity (LVE) region was determined with an oscillatory amplitude sweep test at a constant frequency of 1 Hz in the upward sweep mode. Thus, the values of the limit of the LVE region were 0.2, 0.2, 0.04, 0.04 and 0.02% for CNF-0, CNF-20, CNF-30, CNF-40 and CNF-50, respectively (Supplementary Materials, Figure S1). The angular frequency sweep tests were carried out in the frequency range of 0.1–100 rad·s^−1^ at a fixed strain within the LVE region.

#### 2.2.5. Wide-Angle X-ray Diffraction Study

The crystalline structures of the regenerated BC were studied with wide-angle X-ray diffraction (WAXD) with a Rigaku SmartLab 3 diffractometer (Rigaku Corporation, Tokyo, Japan) equipped with a CuK_α_ radiation source (λ = 1.54 Å) within the 2θ range of 5°–35° with the scan step of 0.05°.

#### 2.2.6. Microscopic Investigation

Atomic force microscopy (AFM) studies were performed using the SPM-9700HT scanning probe microscope (Shimadzu, Kyoto, Japan). The instrument operated in the tapping mode under atmospheric conditions, and NSG10 silicon tips (TipsNano) with curvature radius of 5 nm were employed. Experimental data were processed using the SPM software v.4.76.1 (Shimadzu, Kyoto, Japan).

#### 2.2.7. Application of ChA/Water for 3D Printing with BC

In the present paper, inks refer to the BC/ChA-based UV-curable compositions for 3D printing. Only CNF-30 and CNF-40 compositions were used as photocurable inks for 3D printing because their suitable rheological characteristics (see Section 3.3) ensure a continuous flow at extrusion. A crosslinking agent, N,N’-methylene bisacrylamide (1 wt% of total monomer weight—acrylate ion), and photoinitiator 2-hydroxy-2-methylpropiophenone (1 wt% of total monomer weight) were dissolved in ChA/water prior to BC being added. A 3D BioScaffolder BS3.2 (GeSiM, Radeberg, Saxony, Germany), equipped with a pneumatic syringe and a conical nozzle with an outlet diameter of 0.58 mm, was used for printing. To remove air bubbles, each ink was centrifuged in a syringe at 4000 rpm for 20 min before use. UV-curing, in all cases, was carried out with a mercury lamp OmniCure S1500 (Lumen Dynamics, Mississauga, ON, Canada) equipped with a filter of 320–500 nm. For each layer, 1 W/cm^2^ of light power density was applied for 10–15 s. One-layered strips (filaments) and multilayered cylinders with 100% infill density were printed.

#### 2.2.8. MD Simulations and Model Systems

All atom MD simulations were performed using GROMACS software [44]. As the most popular and widely used option for IL simulations [45,46], the GAFF force-field [47] was used. To take into account electrostatic interactions, atomic partial charges were calculated using the quantum-chemical Hartree–Fock method and the 6-31+G(d,p) basis using the GAUSSIAN16 software package [48]. The charge distribution was calculated using the RESP algorithm [49], according to the recommendations for the GAFF force field [47]. The long-range electrostatic interactions were computed by using the particle-mesh Ewald (PME) method [50,51]. The bond lengths of the hydrogen atoms were constrained with the use of the P-LINCS algorithm [52]. To maintain constant temperature and pressure the Nosé–Hoover thermostat [53,54] and the Parrinello–Rahman barostat [55] were applied. The TIP3p water model [56] was used to describe the behavior of water molecules. The time step was 1 fs in all MD simulations.

MD simulations were performed for two types of systems: (1) the simulation cell containing pure IL and IL with added water, and (2) the simulation cell containing IL (pure and with added water) and the cellulose crystal layer. The simulated IL systems consisted of 600 choline cations, 600 acrylic anions and a number of water molecules corresponding to the water content (0, 10, 20, 30, 40 and 50 wt%) in the ChA/water mixture (see Supplementary Materials, Table S1). The crystalline layer of cellulose consisted of 64 chains containing 16 glucose residues. Periodic boundary conditions ensured the infinite dimensions of the crystal surface.

Because the simulated IL must be very viscous at room temperature to produce properly balanced systems, the simulations were executed from the state of molecular gas pre-equilibrated at 500 K followed by stepwise (25 K) cooling (executed by 50 ns) to 300 K with a final equilibration for 200 ns. To prepare the IL + cellulose systems, the IL samples pre-equilibrated at a high temperature were placed in the common simulation cell with the cellulose layer with further stepwise cooling to 300 K and final equilibration for 200 ns. The structure characteristics were calculated during the last 100 ns. Detailed description of the preparation of the model systems are given in Supplementary Materials (Section S1.2, Figure S2).

For the validation of the IL model, the comparison of the IL densities obtained at different temperatures and water concentrations with experimental data was performed. It is known that in the simulations with the fix-charge force field incorrect results for the systems with ion–ion interactions could be obtained [57,58,59,60]. If such a problem occurs, then it is usually solved by the scaling of charges on the molecules participating in ion–ion interactions [61,62,63]. We considered scaling factors from 0.5 to 1.0 (the full RESP charges) to cover the whole possible range of the charge scaling [61] to find out the best option. Overall, the scanned scaling parameters included 0.5, 0.6, 0.75 and 1.0. The scaling parameter that allowed the best agreement between simulation and experiment was considered the best solution. It turned out that for the IL without added water, the simulations with the full RESP charges gave a very large (about 17%) discrepancy with the experiment, and only using the scaling factor 0.5 led to minimal deviation from the experiment (3.9%) (Supplementary Materials, Table S2). The simulations for the IL with water were performed by using the scaling factor 0.5. More detailed information for the densities of pure IL and for IL with water is given in Supplementary Materials in Tables S2 and S3 and in Figure S3.

#### 2.2.9. Mechanical Measurements

Tensile and compression tests were performed on an Instron 5943 universal testing machine (Instron, Norwood, MA, USA). For uniaxial tensile tests with a traverse speed of 10 mm/min, strips with a gauge length of 20 mm were used. The cross-section of each strip was measured using an optical microscope Altami 104 (Altami, Saint Petersburg, Russia), equipped with a 1.3 MP digital camera VEC-135 (EVS, Saint Petersburg, Russia) and recorded micrographs were processed with ImageJ software v.2.0.0 (National Institutes of Health, Bethesda, MD, USA). Statistical analysis was carried out based on measurements of 5 samples. The compression tests at deformation speeds of 5 mm/min were performed for 3D printed cylindrical-shaped samples with a height of 7–10 mm and diameter of 11–12 mm. The Young’s modulus was calculated from the slope of stress–strain curves in the initial linear region.

## 3. Results and Discussion

### 3.1. Wide-Angle X-ray Diffraction Study

**Figure 1:**
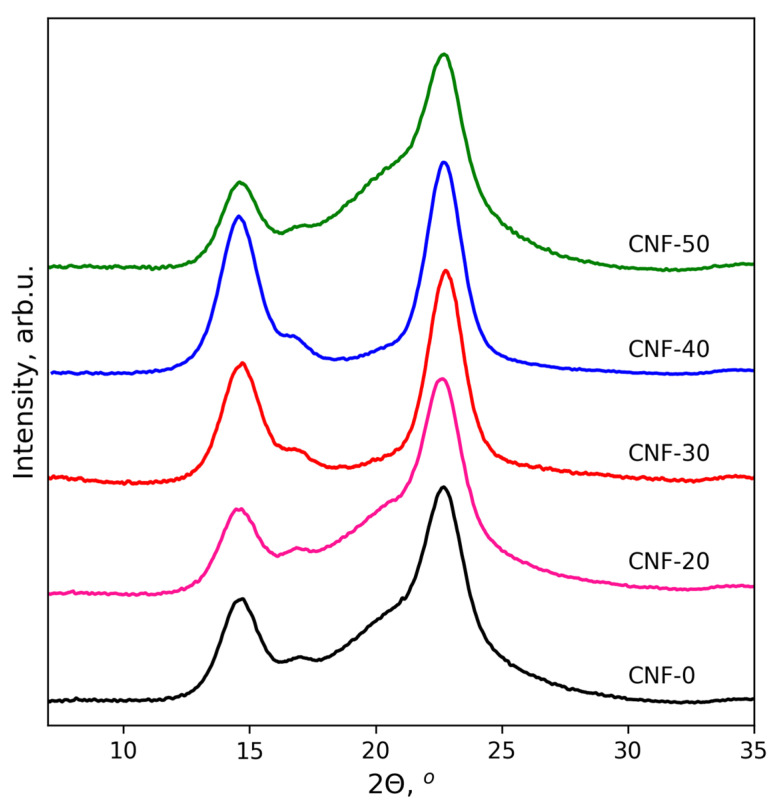
X-ray diffraction patterns for BC nanofibers regenerated from their dispersions in ChA/water mixtures containing 0 (CNF-0), 20 (CNF-20), 30 (CNF-40) and 50 (CNF-50) wt% of water.

The X-ray diffraction study was performed for the BC nanofibers regenerated from dispersions in ChA/water mixtures by the introduction of dispersion into an excess of water. The obtained patterns are given in Figure 1. In the WAXD patterns of all samples, three peaks centered at 2θ = 14.5°, 16.7° and 22.7° are visible. They are typical for cellulose produced by bacteria that represents a mixture of I_α_ and I_β_ allomorphs [64], and can be assigned to the (100), (010) and (110) crystallographic planes of the triclinic unit cell (cellulose I_α_) or the (1–10), (110) and (200) planes of the monoclinic unit cell (cellulose I_β_) [65,66,67]. The crystallinity index (CrI) of regenerated BC nanofibers was calculated according to the Segal method [68]. CrI was 77, 77, 90, 91 and 77% for CNF-0, CNF-20, CNF-30, CNF-40 and CNF-50 samples, respectively. Thus, increasing the water content from 0 to 40 wt% resulted in an increasing degree of cellulose crystallinity. Comparing the obtained crystallinity with that for initial BC (CrI = 96% [43]), an amorphization of cellulose in dispersions without water could be proposed that was in agreement with previously reported results [43]. Increasing the water content led to the diminishing of amorphization, which was visible from the decrease in intensity of the typical broad amorphous halo of cellulose centered near 19.5° [69]. The CrI increased from 77 to 91% for CNF-0 and CNF-40 samples, respectively. However, this trend turned to the opposite direction at the further increase of the water content to 50 wt%. Sample CNF-50 demonstrated a lower degree of crystallinity in comparison with CNF-40: 77%. Thus, the highest degree of crystallinity in the cellulose nanofibers (lowest amorphization) was observed for samples CNF-30 and CNF-40.

### 3.2. Microscopic Investigation

**Figure 2:**
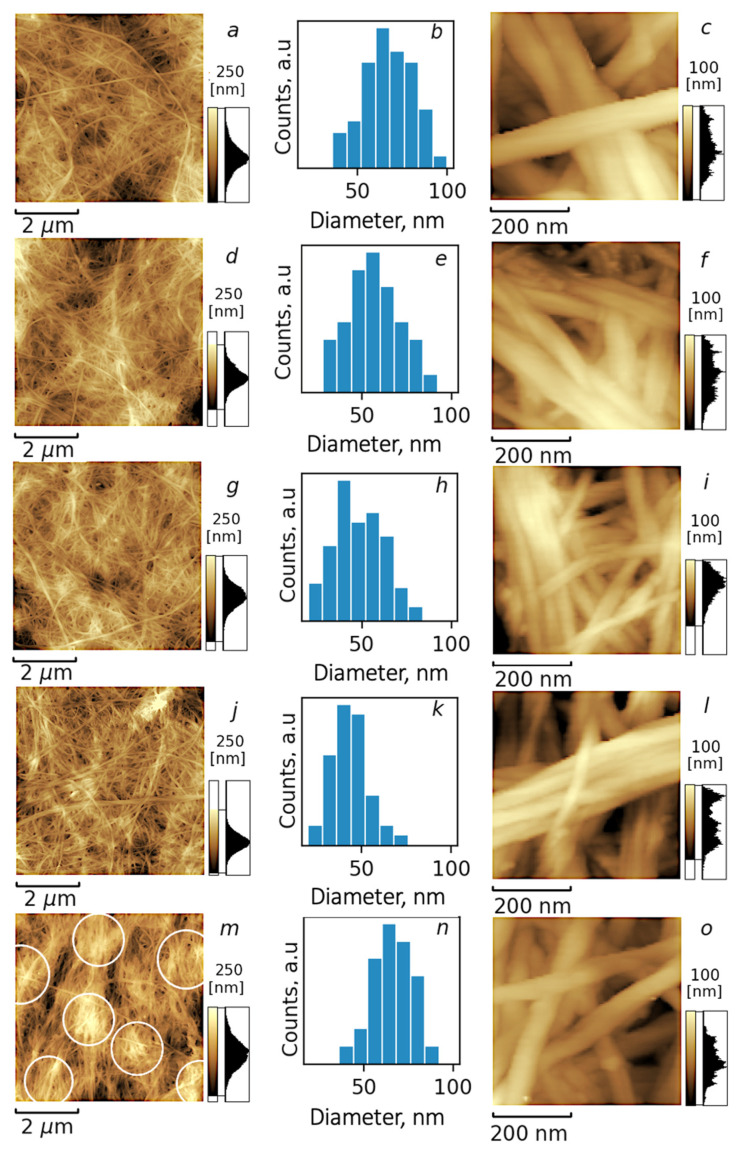
AFM images (**a**,**c**,**d**,**f**,**g**,**i**,**j**,**l**,**m**,**o**) and histograms of diameter distribution (**b**,**e**,**h**,**k**,**n**) of BC nanofibers regenerated from their dispersions in ChA (**a**–**c**) and ChA/water: CNF-20 (**d**–**f**), CNF-30 (**g**–**i**), CNF-40 (**j**–**l**) and CNF-50 (**m**–**o**).

The morphology of BC nanofibers after treatment with ChA/water systems with different compositions were investigated with AFM. The topography maps of regenerated BC are given in Figure 2. It can be seen that in all cases the fibrillar morphology of BC was preserved. However, some differences between samples can be noticed. As was mentioned earlier, the treatment of BC with ChA led to some amorphization of BC that could be attributed to the swelling of CNF under the influence of IL. The analysis of BC nanofiber diameters in the current study was carried out by plotting distribution histograms (see Figure 2b,e,h,k,n for samples CNF-0, CNF-20, CNF-30, CNF-40 and CNF-50, respectively). For the system without water, the increased diameter of CNF can be attributed to the strong interaction between cellulose and Ils, leading to the amorphization of their surface. The decrease in the diameter of CNF by increasing the water content up to 40 wt% is connected with the hydration of IL ions, which hampers their interaction with cellulose. When the water content is further increased to 50 wt%, the formation of clusters with free water is significantly enhanced as it will be demonstrated further with MDs simulation. It can be hypothesized that this free water contributes to the swelling of CNF at the highest water content. The possibility of CNF swelling in water was directly demonstrated in the literature [70], which supports our hypothesis. It also should be mentioned that the formation of a hydration shell on CNF also can influence the measured diameter. The amorphization of the CNF surface can enhance its water sorption ability, increasing the thickness of the hydration shell. Thus, it should be taken into account that the hydration of swelled CNF at ambient conditions also contributes to the observed maximal CNF diameter at 0 and 50 wt%. It was also observed that increasing the water content up to 50 wt% led to the formation of seals from several cellulose fibers, as is shown in Figure 2 with white circles. It should be noted that during measurements of the diameter we were focused on the dimension of individual nanofibers. Thus, agglomeration does not influence the presented distributions of diameters. Thus, the minimum of swelling can be proposed for samples containing 30 and 40 wt% of water that are in agreement with WAXD data.

### 3.3. Rheological Properties of BC Nanofiber Dispersions in ChA/Water

**Figure 3:**
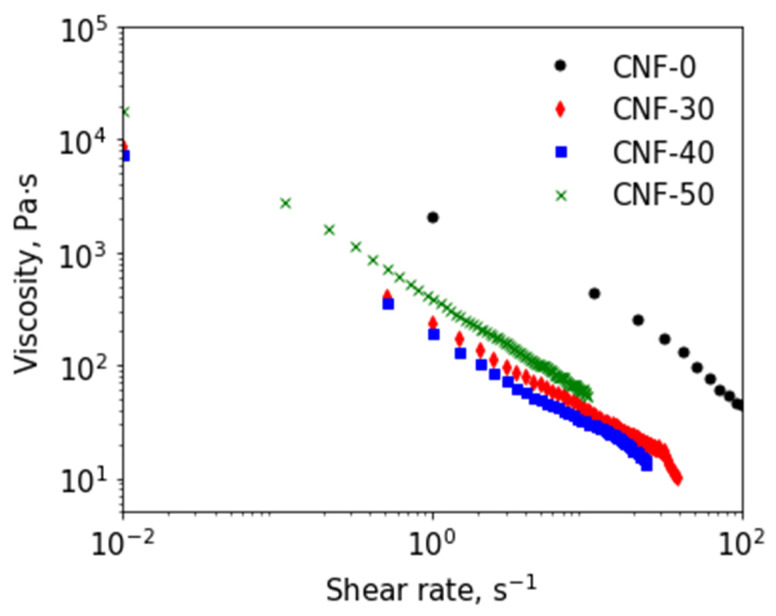
Dependence of viscosity on shear rate for dispersions of BC nanofibers in ChA and ChA/water.

**Figure 4:**
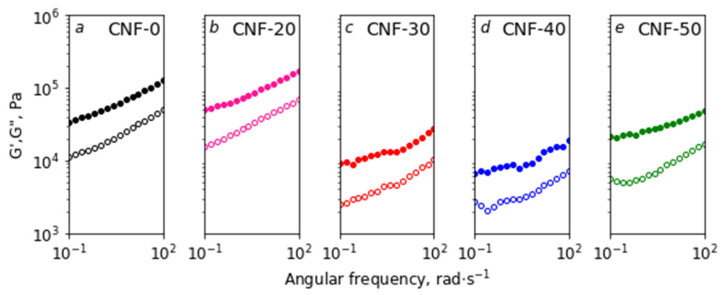
Storage (G′, filled symbols) and loss (G″, hollow symbols) moduli as a function of angular frequency for dispersions of BC nanofibers in ChA and ChA/water.

The rheological properties of BC nanofiber dispersions were studied in steady shear and oscillation modes. Experimental curves are given in Figure 3 and Figure 4, respectively. The yield stress (τ_y_) for all dispersions was determined from oscillatory amplitude sweep tests (see Supplementary Materials, Figure S4). Viscosity curves (Figure 3) demonstrated that all dispersions behaved like non-Newtonian liquids. The highest viscosity value was observed for anhydrous composition CNF-0. This was in agreement with the yield stress value of 2230 Pa for this sample, which was found to be maximal among all investigated dispersions (Figure S4a).

The addition of 20 wt% of water to the dispersion led to a decrease in yield stress: from 2230 to 1280 Pa for CNF-0 and CNF-20, respectively. Both τ_y_-values were too high to use these compositions (inks) for 3D printing with reasonable pressure. Moreover, the CNF-20 dispersion demonstrated a tendency to eliminate the liquid solvent and phase separation that became more intensive under shear stress. This hampered the measurement of proper flow curves for this sample. Thus, the CNF-0 and CNF-20 dispersions were not used for 3D printing. As can be seen in Figure 3, a further increase in the water content in the mixed solvent (up to 30–40 wt% of water) led to a viscosity decrease by nearly an order of magnitude in the whole range of applied shear rates. This was in agreement with decreasing yield stress being 160 and 97 Pa for samples CNF-30 and CNF-40, respectively (see Figure S4c,d). Moreover, in the water content range of 30–40 wt%, the elimination of liquid or phase separation was not observed. Thus, the physico-chemical properties of the CNF-30 and CNF-40 make them suitable for 3D printing. In the case of the CNF-50 sample, the shear viscosity was higher in comparison than that for CNF-30 and CNF-40, but lower than that for the CNF-0 (see Figure 3). The yield stress for the CNF-50 was 374 Pa (Figure S4e), which was two times higher than for the CNF-40, indicating a more rigid structure of the CNF-50 that could be the consequence of CNF agglomeration observed with AFM. At the same time, the CNF-50 dispersion was not stable under load because of phase separation, which makes it inappropriate for 3D printing.

The results of the rheological study into the oscillating mode are shown in Figure 4. It can be seen that the dynamic storage moduli (G′) for all the dispersions exceeded the loss moduli (G″), which corresponds to the gel-like behavior with defined yield stress. The monotonous reduction of modules with decreasing frequency allowed us to attribute them to “weak” gels, which are caused by the physical nature of crosslinks [71]. Curves for the CNF-0 and CNF-20 samples were approximately equal (Figure 4a,b). The sharp decrease in G′ and G″ was observed with an increase in water content from 20 to 30 wt% (Figure 4b,c). Some increase in G′ and G″ was visible when moving from the sample CNF-40 to CNF-50 (Figure 4d,e). It could also be noticed that, in the case of the CNF-50 sample, the minimal value of loss tangent (tanδ = G″/G′) was observed (see Supplementary Materials, Figure S5). This indicates the more pronounced, solid-like behavior of the CNF-50 sample in comparison with others [72], and also confirms CNF agglomeration.

Summing up the discussion of the results of rheological measurements, the following factors affecting the rheological behavior of gels can be noted: (1) the interaction between CNF (interfibrillar interaction); (2) the interaction between CNF and the solvent (dispersion media); and (3) the viscosity of the solvent. Amorphysation and swelling on the surface of CNF increases the amount of accessible OH groups that can interact with surroundings and enhances the friction between the fibers inside the dispersion during shear stress. This factor, along with the higher viscosity of IL in comparison with water, hampers the 3D printability of samples with low water content (0 to 20 wt%). Increasing the interfibrillar interaction results in the growing of viscosity and yield stress. This was observed when water content increased from 40 up to 50 wt%, as can be seen from the results of rheological measurements. This can be attributed to the fact that the highest studied water content (50 wt%) resulted in the coagulation of cellulose and the formation of agglomerates, which was demonstrated with AFM earlier.

The energy of solvent interaction with OH groups on the surface of CNF determines the stability of dispersion. Increasing this energy leads to the decrease in interfibrillar interaction and should result in a decrease in the yield stress and viscosity of dispersion. However, increasing IL content from 60 to 100 wt% (backwards from sample CNF-40 to CNF-0) results in the growth of yield stress. In the case of ChA/water mixtures, the significantly higher viscosity of IL in comparison with water should be taken into account (0.77–3.58 Pa·s for ChA [40] and 8.90·10^−4^ Pa·s for water [73]). Decreasing the water content leads to the significant growth of solvent viscosity due to the formation of a strong network of ionic interactions in IL that also affect the rheological behavior of studied systems.

### 3.4. Molecular Dynamics Simulations

#### 3.4.1. IL Structure and Effect of Water

**Figure 5:**
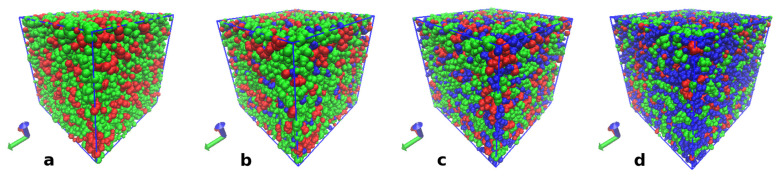
Snapshots of the IL equilibrated systems: S-0 (**a**), S-10 (**b**), S-30 (**c**) and S-50 (**d**). Choline cations are represented by a green color, acrylic anions are represented by a red color and water molecules by a blue color. Visualization was performed using the Visual Molecular Dynamics (VMD) software [74].

Examples of the IL equilibrated structures are shown in Figure 5.

The main structural features of IL and the interactions responsible for the structure formation, as well as the effect of the added water, were studied. The radial distribution functions (RDF, see Equation (S1)) between the atoms involved in the ion–ion, ion–dipole and dipole–dipole interactions were analyzed. RDFs were calculated for the following pairs of atoms: oxygens of acrylic anion and nitrogens of choline cations, as well as hydrogens of choline hydroxyl groups (O_A_ and N_Ch_ as well as O_A_ and HO_Ch_) for pure IL. For the systems with water, the following pairs were additionally considered: water hydrogens (H_H2O_) with O_A_ and O_Ch_, and also water oxygens (O_H2O_) with HO_Ch_. Water–water RDFs (O_H2O_-H_H2O_) were calculated also. The RDFs for the atom pairs of the choline and acrylic ions are presented, as an example, in Supplementary Materials (Figure S6). To estimate the different interactions responsible for the IL structure formation, the potentials of the mean force (PMF) were calculated by using RDF (Equation (1)):

(1) $$PMF=-RTln(g\left(r\right))$$

where *R* is the universal gas constant (8.314 J/mol∙K), *T* is the temperature (300 K in this simulation), and *g*(*r*) is the RDF.

**Figure 6:**
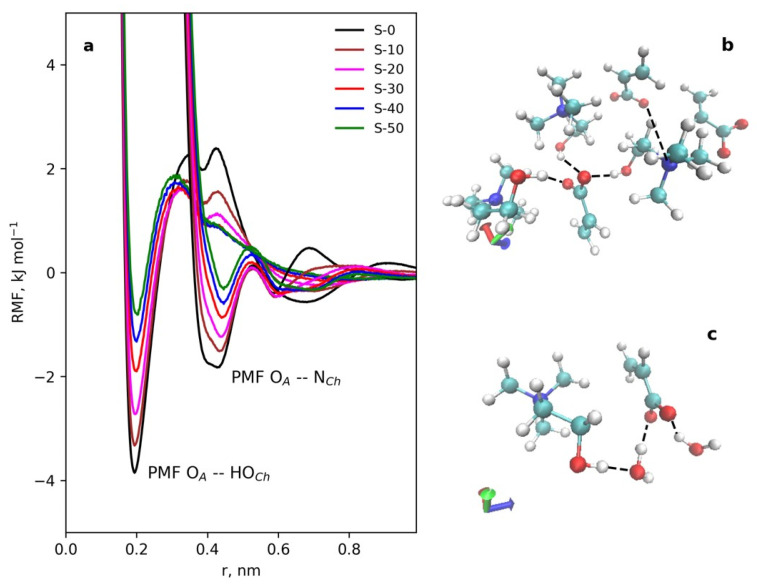
The PMFs for the main interactions: ion–dipole interactions (O_A_ with O_Ch_) and ion–ion interactions (N_Ch_ with O_A_) in pure IL and with added water (**a**). A snapshot of a typical coordination of IL molecules involved in the ion–ion interactions and in the ion–dipole interactions (**b**). A snapshot of typical contacts of water molecules with IL components (**c**). Atoms colors are as follows: carbon: cyan, oxygen: red, hydrogen: white, nitrogen: blue. The dotted lines indicate coordination bonds. Visualization was performed using the VMD software [74].

Figure 6a illustrates the PMFs for the main interactions, ion–dipole interactions (O_A_ with O_Ch_) and ion–ion interactions (N_Ch_ with O_A_), and their changes with water concentration. In Figure 6b,c a fragment of the pure IL structure and a fragment of the structure with water (S-30) are shown, respectively.

**Figure 7:**
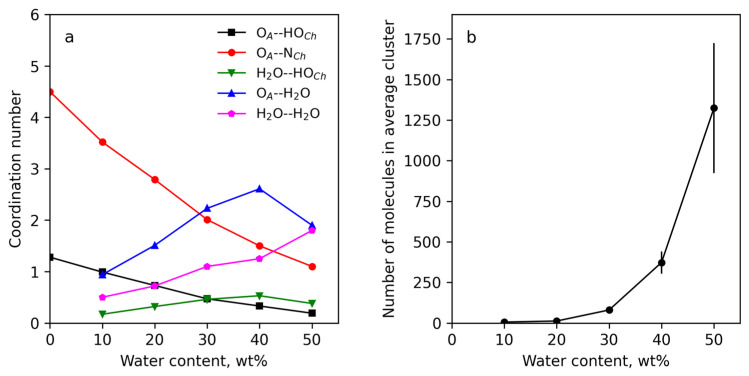
The coordination numbers of the choline cation active atoms near the acrylic anion oxygens and water hydrogen and oxygen atoms near acrylic anion and choline cation vs. added water (**a**). Average size of water cluster vs. added water (**b**).

The main stabilizing factor of the IL structure was the ion–dipole interaction (O_A_ with HO_Ch_), while the ion–ion interaction was twice as weak because CH_3_ groups of choline cation hinder the ion–ion close contact. The interactions between IL components became weaker with the increase in water concentration due to their shielding by H_2_O molecules attracted by choline and acrylic ions. Using the RDFs, the coordination numbers (the amounts of molecules in the first coordination shell of the referenced molecules) were calculated by using Equation (S2) (see Supplementary Materials). The results are presented in Supplementary Materials (Figure S7). These data were collected in Figure 7 as the coordination numbers vs. added water. The analyses of the dependences showed that the amount of contact between the IL components decreased with water concentration, and acrylic anions were the main water-absorbing component. The data in Figure 7a evidenced the non-monotonic changes in the contacts of the IL components with water molecules with the increase in water concentration. With up to 40 wt% of added water, the number of the contacts increased, but then their number fell. This meant that there was a limit to the water molecules’ absorption by IL, and at water concentrations above the limit water molecules prefer to interact between each other. This can be seen from the changes in the water–water coordination number (Figure 7a), which achieved the value 2 for the S-50 sample. In other words, each water molecule coordinated with 2 water molecules, forming an H-bonds network. This conclusion is confirmed by the result of the cluster analysis (Figure 7b, average cluster size vs. water concentration), showing the association of water molecules into huge clusters at 50 wt% water concentration.

Concluding the MD simulation of ChA/water solvent, it can be postulated that with increasing water concentration the interactions between the IL components decreases, which obviously should lead to a decrease in the viscosity of the solvent. Moreover, there is a limiting concentration of water above which the amount of water bound by the IL decreases and a portion of free water increases.

#### 3.4.2. Structure of Cellulose Near-Surface Layer and Water Influence

**Figure 8:**
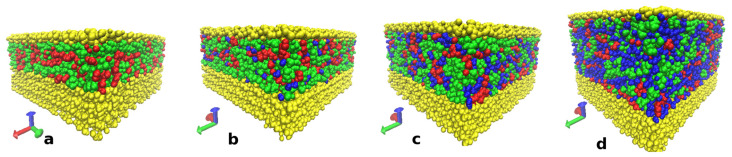
Snapshots of the ChA/water/BC equilibrated systems for CNF-0 (**a**), CNF-10 (**b**), CNF-30 (**c**) and CNF-50 (**d**). Choline cations are represented by green, acrylic anions by red, water molecules by blue and cellulose molecules by yellow. Visualization was performed using the VMD software [74].

The snapshots of the equilibrated systems of ChA/water/BC are shown in Figure 8.

**Figure 9:**
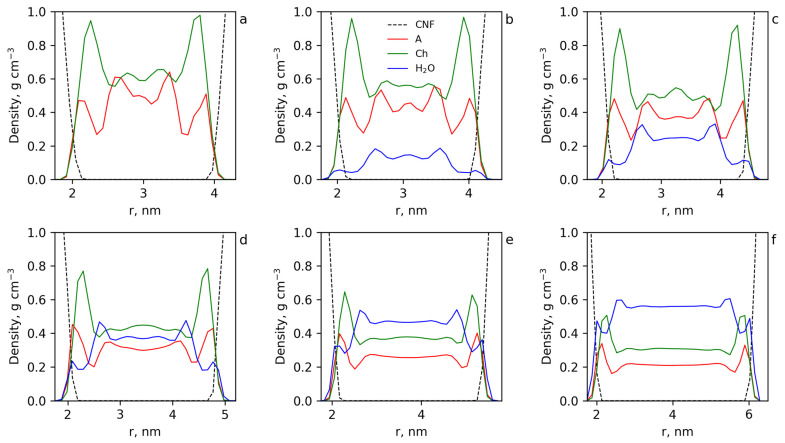
The mass density profiles of the choline (green), acrylate (red), water (blue) and cellulose (black) for the cases CNF-0 (**a**), CNF-10 (**b**), CNF-20 (**c**), CNF-30 (**d**), CNF-40 (**e**) and CNF-50 (**f**).

To find out how the IL components and water were distributed at the cellulose surface and in the bulk of the solvent, the mass density profiles were calculated (Figure 9).

It can be seen that for the system without water (Figure 9a), both acrylic anions and choline cations were adsorbed by the cellulose surface in an equivalent amount (taking into account the difference in their molecular masses). With the increase in water concentration, the amount of water molecules at the cellulose surface increased, partly displacing choline and acrylic ions. Figure S8 (see Supplementary Materials) shows a fragment of the cellulose molecule with the atoms participating in the interactions of the cellulose surface with acrylic anion, choline cation and water atoms numbered. To clear up how the IL components and water interact with the cellulose surface, the coordination numbers (Figures S9 and S11) as well as PMFs (Figures S10 and S12) for the main interacting atoms on the cellulose surface (hydrogen and oxygen atoms of the primary and secondary hydroxyl groups and oxygen atoms of the glucose cycles and between them) with the mentioned active atoms of choline cation, acrylic anion and water were calculated. The results for the coordination numbers for the system without water are presented in Figure S9. The data demonstrated that the hydroxyl groups at C6 and C2 atoms of the glycoside ring that were most transposed into solution had the largest number of contacts, with the IL components. The analysis of the PMFs for these interactions (Figure S10) showed that the main contribution to the adsorption of the IL components to the cellulose surface gave the energetically favorable interaction of acrylic anions (Figure S10a), and choline cations were involved in the near-surface layer mainly indirectly by acrylic anions. This conclusion is based on the analysis of the PMF for choline interactions with the active groups of the cellulose surface (Figure S10b) demonstrating the unprofitability of these contacts, but since the cations nevertheless occur near the surface, the unprofitability of their contacts with the surface probably is compensated for by the gain from the interactions of choline cations with acrylic anions.

**Figure 10:**
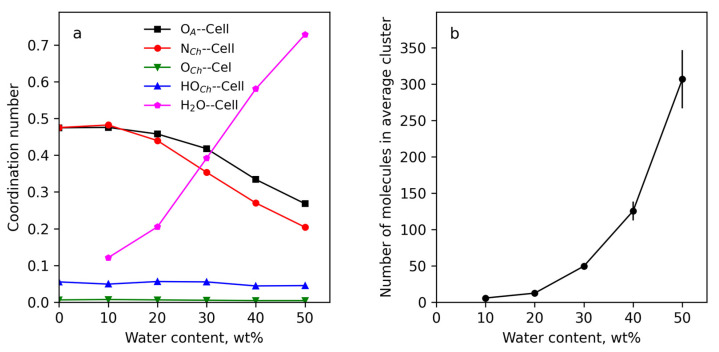
The numbers of the contacts of water molecules and IL components per cellulose monomer (**a**). Number of water molecules in the average water cluster vs. water concentration (**b**).

The coordination numbers for the active groups and atoms on the cellulose surface (hydrogen and oxygen atoms of the primary and secondary hydroxyl groups of cellulose and oxygen atoms in glucose cycles and between them) with oxygen and hydrogen atoms of water were also calculated (Figure S11). The data show that water molecules were mainly in the close vicinity of hydroxyl groups at C6 and C2 atoms, which were also the adsorption centers of acrylic anions on the cellulose surface. Taking into account the energy unfavorability of water contacts with the cellulose surface and the advantageousness of the water interaction with acrylic anions (Figure S12a,b) it can be concluded that water molecules penetrate to the cellulose surface to realize their contacts with acrylic anions. The numbers of the contacts of the IL components and water per cellulose monomer at each water concentration were calculated by summing the coordination numbers for each pair of the active atoms of the surface cellulose molecules with the active atoms of choline cations, acrylic anions and water molecules (Figure S13). The dependencies of the integral contacts on water concentration are presented in Figure 10a and demonstrate a strong decrease of the contacts of the IL components with the cellulose surface with water concentration > 30 wt%. This result explains the sharp decreasing yield stress at the water concentration step from 20 to 30 wt%. The number of water molecules occurring near the surface cellulose molecules strongly increases with water concentration. However, the complete suppression of contacts of the IL with cellulose is not observed even at 50 wt% water content (Figure 9).

The results of the cluster analysis (Figure 10b) show that water molecules formed much smaller clusters than in the systems without cellulose. This is due to water penetrating to the cellulose surface and accumulating at the surface of the cellulose in a significant amount (Figure 8d).

The PMFs for the interactions of water molecules with the active groups on the cellulose surface (Figure S12b) show that these interactions are unfavorable for the system. The driving force for the accumulation of water molecules at the surface of cellulose is the advantageous interaction of water molecules with acrylic anions interacting with the surface, and the benefits of both interactions are comparable (see Figures S10a and S12a). Thus, water molecules compete with the active groups of cellulose for interactions with the acrylic anions and screen them from the surface, forming a hydration shell. At high water concentrations the interactions of water molecules with each other begin to play an important role, which leads to the formation of water clusters in bulk and at the cellulose surface.

The presented data set of MD simulation allows us to propose the molecular mechanisms explaining experimental properties and structural features of CNF dispersions in IL/water mixtures. The diminishing amorphization of CNF with increasing water content can be explained by the intensive water bonding to the components of IL, thus preventing their interaction with cellulose. This effect reaches a maximum at 40 wt% of water and decreases when the water content is increased up to 50 wt%. When the water is increased up to 50 wt%, the free water appears and the number of IL-water contacts decreases—a solvent becomes heterogeneous on the nano level. Thus, the local intensity of IL interaction with cellulose can be enough for starting cellulose swelling, while free water, sorbing on the new forming amorphous cellulose parts, can accelerate this process. As a result, the stability of CNF dispersion at water content > 40 wt% decreases and agglomeration was observed visually and with AFM.

### 3.5. Mechanical Properties and 3D Printing

**Figure 11:**
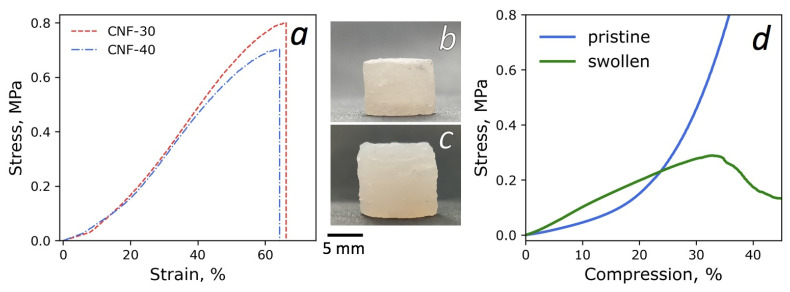
Typical tensile stress–strain curves measured in tensile mode for dried composites after polymerization (**a**); photographs of 3D printed cylindrical-shaped samples based on CNF-30 composition before (**b**) and after being immersed in saline solution (**c**); and typical stress–strain curves measured in compression mode (**d**).

The mechanical properties of the 3D-printed photocured composites were investigated. Strip samples based on the CNF-30 and CNF-40 were produced by 3D printing. For the best comparison, the effect of water in the samples was eliminated by drying in a vacuum oven at 60 °C before the experiment. Typical tensile stress–strain curves for the samples are presented in Figure 11a. It was found that the increase in water content from 30 to 40 wt% in the dispersion led to a slight decrease in the average tensile strength (*σ_r_*) from 0.8 to 0.7 MPa, and in Young’s modulus (*E*) from 1.2 to 1.1 MPa. Nevertheless, the *σ_r_* and *E*-values were within the statistical error limit of ±0.2 MPa and ±0.3 MPa, respectively. This correlates with a decline in the yield stress from 160 Pa to 97 Pa for the dispersions, containing 30 and 40 wt% of the water, respectively. Notably, *σ_r_* and *E*-values for the obtained composites reinforced with CNF were higher than those for the unfilled polymerized deep eutectic solvent (AA/ChCl 2:1 mol:mol), which exhibited *E* of ~0.25 MPa and *σ_r_* of ~0.3 MPa [75].

BC/ChA-based inks are considered to be promising for the 3D printing of various complex-shaped constructs. For example, composite hydrogels based on synthetic polymers (polyacrylic acid, polyacrylamide) are currently being actively studied for medical applications such as scaffolds for bone tissue engineering [76] and cartilage substitutes [77]. In this regard, two types of 3D-printed cylinders were obtained based on CNF-30 dispersion: (1) a sample containing 30 wt% of the water after photocuring, i.e., without drying prior to mechanical measurements, and designated as a “pristine” sample in the following text; and (2) a sample that was kept in a physiological saline solution (0.9 wt% NaCl) for 12 days to mimic the in vivo osmotic conditions and designated as “treated” sample. The mechanical properties of the specimens were investigated in compression and the corresponding typical stress–strain curves are presented in Figure 11d. The pristine sample showed an exponential increase in stiffness, whereas the swollen sample demonstrated a relatively linear response up to ~30% compression. A nonlinear behavior under loading is characteristic of the cross-linked polymer hydrogels and most soft biological tissues [78]. As seen from Figure 11d, the stress values for the swollen specimen exceeded the pristine one at the low-strain region. The compression modules (*E*_c_) defined in the linear region of 0–5% were 0.83 MPa and 0.40 MPa for swollen and pristine hydrogel, respectively. However, at compression > 25% the stress in the pristine sample became higher than in the swollen one. The compressive stress at break (*σ_c_*) for the swollen sample was found to be 0.26 MPa, which was lower than the value of compression stress (0.65 MPa) for the pristine sample at the deformation of 35%. The difference in the mechanical behavior between swollen and pristine samples can be explained by the following. In the pristine sample, the interaction between poly-IL and CNF led to the existence of some mobility of CNF under load and, as a result, the system behaved as an elastic cross-linked network. In the case of the sample treated in water (swollen), the hydration of choline ions and carboxylic groups of polyacrylic acid inhibited their interaction with CNF. As a result, cellulose nanofibers moved closer to each other and their possible aggregation was facilitated in the swollen sample, similar to what was observed for diluted dispersion CNF-50 before UV-curing (see Section 3.2). The formation of CNF-CNF contacts may result in a more rigid and brittle structure [79].

The strong agglomeration of CNF can be proposed during treatment in aqueous NaCl due to interfibrillar hydrogen bonding. This results in inelastic, solid-like behavior. It should be noted that both reinforced hydrogels (swollen and pristine) demonstrate higher mechanical characteristics in compression mode than the hydrogel based on copolymerized poly(ethylene glycol) diacrylate and choline acrylate (*E*_c_ = 89.8 kPa, *σ_c_* = 0.16 MPa). This allows the postulation of a significant reinforcing effect of CNF in both cases.

## 4. Conclusions

In this work, the effect of water as a co–solvent on the interaction between polymerizable ionic liquid (IL), choline acrylate (ChA) and bacterial cellulose (BC) was studied. The measurement of rheological characteristics showed that for an anhydrous system (BC/ChA), the yield stress value was large for 3D printing (2230 Pa). The introduction of water made it possible to reduce yield stress due to the decrease of both the number of interactions between ions forming IL and between IL and cellulose. It was demonstrated with molecular dynamics (MD) simulations that the hydration of acrylic ions was the highest for systems containing 30–40 wt% of water. When further increasing the water content, the significant impact on the energy system has contacts between water molecules. MD results allow the proposition of the optimal IL/water ratio for 3D printability being connected with the situation when the ion–dipole interactions between IL components and cellulose-IL in ink are depressed to some extent by water, while the number of contacts between cellulose and IL components are higher than contacts between water and cellulose per monomer unit. This appears to be enough for the stabilization of dispersion, but provides the optimal intensity of interaction for the preservation of cellulose nanofibers (CNF)’s morphology and crystalline structure. Additionally, the water–acrylate interaction with the formation of complexes led to the destruction of a network of ionic bonds between IL cation and anion and decreased the viscosity of dispersion.

Thus, inks containing 30–40 wt% of water demonstrate a suitable yield stress for 3D printing and the highest degree of crystallinity and the lowest swelling of CNF. At a water content of 50 wt% agglomeration of CNF, their amorphization and swelling was observed. Additionally, yield stress for systems with the highest water content begins to increase. Thus, the optimal composition of ink based on polymerizable IL (ChA) and CNF for 3D printing was found and successfully used for test 3D printing.

## Data Availability

Not applicable.

## Supplementary Materials

The following supporting information can be downloaded at: https://www.mdpi.com/article/10.3390/polym15092156/s1, Figure S1: Storage (G′, filled symbols) and loss (G″, hollow symbols) moduli as a function of strain for dispersions; Table S1: List of the number of water molecules added to IL systems and the volumes of the simulation cell of the IL systems after equilibration; Figure S2: Snapshot of the starting configuration for the IL + cellulose system. The IL equilibrated cell is shown for CNF-30. Choline cations are represented by green, acrylic anions by red, water molecules by blue and cellulose molecules by yellow; Table S2: The simulated densities of the IL without water for the different values of charge scaling and the experimental data (300 K); Table S3: The experimental and the simulated (for charge scaling 0.5) densities of the IL with water at 300 K and the deviations between them; Figure S3: Comparison of densities between experiments and MD simulations with charge scaling coefficient 0.5 for S-0, S-10, S-20, S-30, S-40 and S-50; Figure S4: Storage (G′, filled symbols) and loss (G″, hollow symbols) moduli as a function of shear stress for dispersions; Figure S5: Dependence of the loss tangent on the angular frequency for dispersions of BC nanofibers in ChA and ChA/water mixtures; Figure S6: Radial distribution functions for the following pairs: HO_Ch_-O_A_ (a), N_Ch_-O_A_ (b); Figure S7: Coordination numbers for the following interactions: HO_Ch_-O_A_ (a), N_Ch_-O_A_ (b), HO_Ch_-H_2_O (c), O_Ch_-H_2_O (d), O_A_-H_2_O (e) and H_2_O-H_2_O (f); Figure S8: Fragment of the cellulose molecule with numbering of the atoms participating in the interactions of the cellulose surface with acrylic anion, choline cation and water atoms; Figure S9: Coordination numbers for hydrogen and oxygen atoms of the primary and secondary hydroxyl groups of cellulose and oxygen atoms of the glucose cycle with oxygen atoms of acrylic anions (a), nitrogen atoms of choline cations (b), oxygen atoms of choline hydroxyl groups (c) and hydrogen atoms of choline hydroxyl groups (d) for the system without water. Oxygen atoms (O1) connecting glucose cycles are not achievable for the IL components and the data for them are not presented; Figure S10: Potentials of mean force for oxygen atoms of the primary and secondary hydroxyl groups of cellulose and oxygen atoms of acrylic anion (a) and for oxygen atoms of the primary and secondary hydroxyl groups and glucose cycles of cellulose with nitrogen atoms of choline cations and with hydrogen atoms of choline hydroxyl groups as well as hydrogen atoms of cellulose hydroxyl groups with oxygen atoms of choline hydroxyl groups (b); Figure S11: The presented dependencies demonstrate amounts of water contacts with active groups and atoms of cellulose monomers. The coordination numbers for hydrogen and oxygen atoms of the primary and secondary hydroxyl groups of cellulose and oxygen atoms in glucose cycles and bridging glucose cycles with oxygen and hydrogen atoms of water were summed for CNF-10 (a) and CNF-50 (b); Figure S12: Potentials of mean force for the interactions of choline cation (oxygen and hydrogen atoms) and oxygen atoms of acryl anions with oxygen and hydrogen atoms of water (a) and hydrogen and oxygen atoms of primary and secondary hydroxyl groups of cellulose and oxygen atoms in glucose cycles and bridging glucose cycles with oxygen and hydrogen atoms of water (b); Figure S13: The numbers of the contacts per cellulose monomer were obtained by summing coordination numbers for hydrogen and oxygen atoms of the primary and secondary hydroxyl groups of cellulose and oxygen atoms of the glucose cycle with nitrogen atoms of choline cations (a), oxygen atoms of acrylic anions (b), oxygen and hydrogen atoms of water (c), hydrogen atoms of choline hydroxyl groups (d) and oxygen atoms of choline hydroxyl groups (e).
