# Supplementary material for: Water Influence on the Physico-Chemical Properties and 3D Printability of Choline Acrylate—Bacterial Cellulose Inks
Source: Polymers (Basel). 2023 Apr 30;15(9):2156. doi: 10.3390/polym15092156 (PMC10181127; doi:10.3390/polym15092156)
Supplement: Supplementary file 1 [file polymers-15-02156-s001.zip › polymers-2346042-supplementary.pdf]

Polymers

ELECTRONIC SUPPLEMENTARY INFORMATION FOR

Water influence on the physico-chemical properties and 3D printability of choline acrylate - bacterial cellulose inks

Veronika S. Fedotova[1], Maria P. Sokolova[1], Vitaly K. Vorobiov[1], Eugene V. Sivtsov [2], Natalia V. Lukasheva, Michael A. Smirnov[1]

[1] Institute of Macromolecular Compounds, Russian Academy of Sciences, V.O. Bolshoi Pr. 31, 199004 St. Petersburg, Russia
[2] Saint Petersburg State Institute of Technology, Moskovsky prospekt 24-26/49, 190013 St. Petersburg, Russia
* Correspondence: pmarip@mail.ru; lukasheva.natalia.v@gmail.com; smirnov_michael@mail.ru.

1. Experimental part

1.1. Determination of the limit of linear viscoelasticity for ChA/water/BC

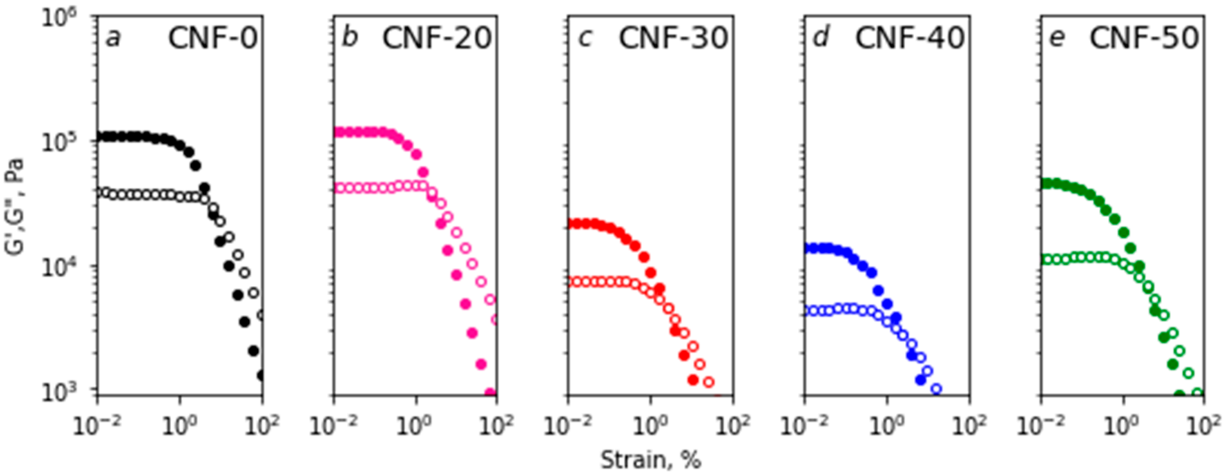

Figure S1. Storage (G′, filled symbols) and loss (G″, hollow symbols) moduli as a function of strain for dispersions.

1.2. Molecular dynamics (MD) simulations

1.2.1. MD simulation procedure and models

Table S1. List of the number of water molecules added to IL systems and the volumes of the simulation cell of the IL systems after equilibration.

N/N	Concentration of water in ChA/water mixture, wt%	Number of water molecules	Simulation cell volumes in nm^3 after equilibration
1	0	0	5.30766^3
2	10	624	5.51114^3
3	20	1317	5.71735^3
4	30	2500	6.05462^3

5	40	3889	6.49232^3
6	50	5833	6.86562^3

To prepare the IL+cellulose systems the pre-equilibrated at high temperature (400 K) the IL samples with 0, 10, 20, 30, 40 and 50 wt% water were placed on the cellulose surface and then each system was balanced in a common simulation cell for cellulose and IL. The example of the starting structure is shown in Figure S3 with the dimensions of the cellulose layer. First, taking into account the large size difference of the IL (the volumes of the IL equilibrated systems are presented in Table S1) and cellulose boxes, the common simulation cell with the cellulose layer and the cube of IL molecules was semi-isotropically compressed at 400 K during 50 ns by a pressure 10 bar to obtain evenly filled space above cellulose. Then the systems were equilibrated during 100 ns at pressure 1 bar and the final configurations were sequentially cooled to 300 K. At 300 K the systems were equilibrated during 200 ns.

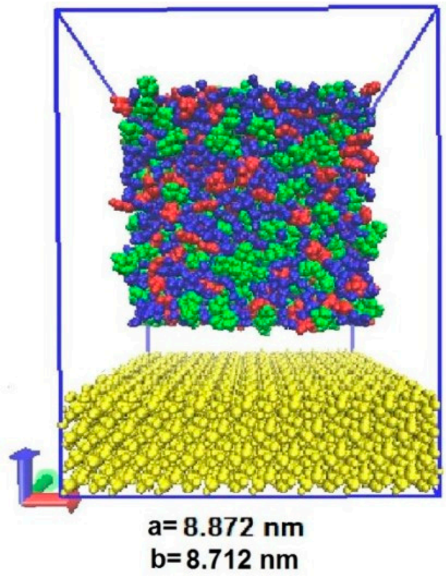

Figure S2. Snapshot of the starting configuration for the IL+cellulose system. The IL equilibrated cell is shown for CNF-30. Choline cations are represented by green, acrylic anions by red, water molecules by blue and cellulose molecules by yellow color.

1.2.2. Force field validation

Table S2. The simulated densities of the IL without water for the different values of charge scaling and the experimental data (300 K).

**Charge scaling**	1	0.75	0.6	0.5	**Experiment**
**Density, kg·m^{-3}**	1310.3	1227.7	1212.4	1167.8	1123.1
**Deviation from experiment in %**	16.6	9.3	7.8	3.9	-------

Table S3. The experimental and the simulated (for charge scaling 0.5) densities of the IL with water at 300 K and the deviations between them.

**Concentration of water in ChA/water mixture, wt%**	10	20	30	40	50
**Density in simulation, kg·m^{-3}**	1158.7	1146.9	1125.0	1103.3	1080.3
**Density in experiment, kg·m^{-3}**	1111.5	1103.7	1093.4	1081.4	1068.5
**Deviation in %**	4.0	3.8	2.8	2.0	1.1

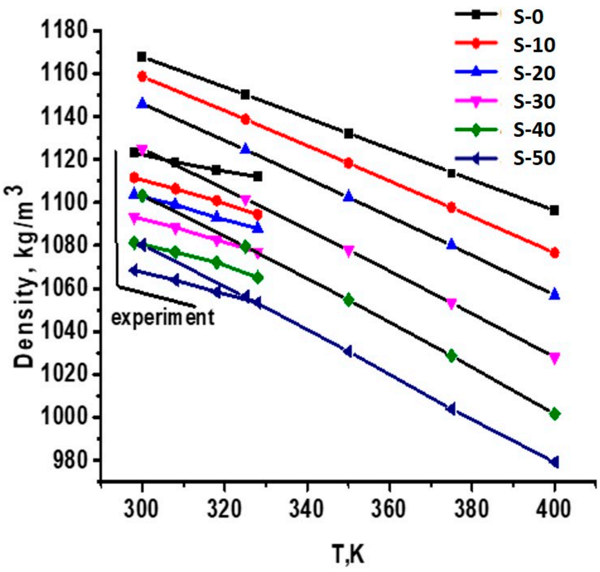

Figure S3. Comparison of densities between experiments and MD simulations with charge scaling coefficient 0.5 for S-0, S-10, S-20, S-30, S-40 and S-50.

2. Results

2.1. Rheological properties

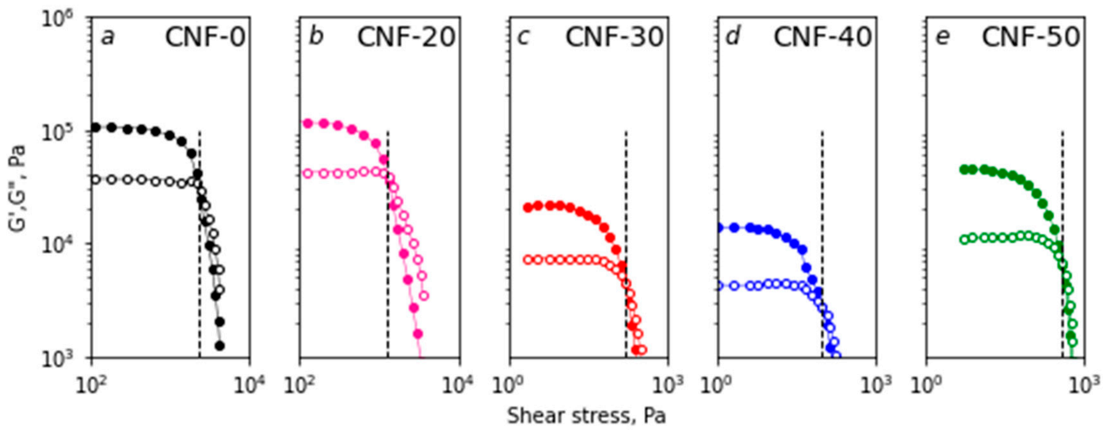

Figure S4. Storage (G′, filled symbols) and loss (G″, hollow symbols) moduli as a function of shear stress for dispersions.

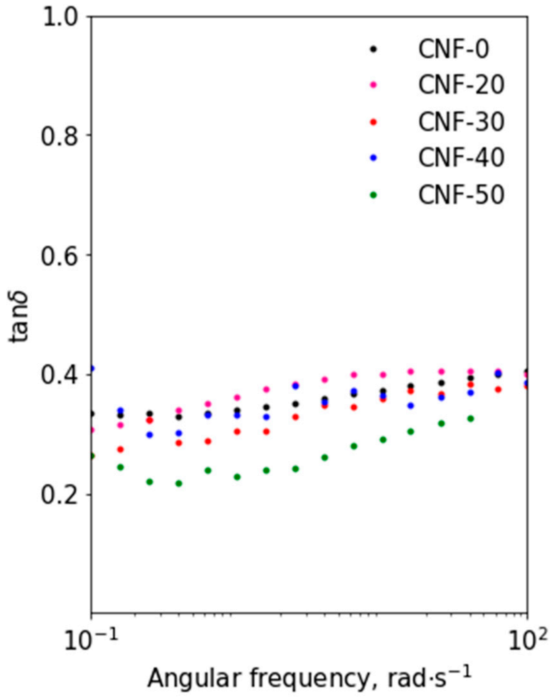

Figure S5. Dependence of the loss tangent on the angular frequency for dispersions of BC nanofibers in ChA and ChA/water mixtures.

2.2. MD simulations

RDFs were calculated using Gromacs' tools as

$$g_{AB}(r) = \frac{1}{N_A \rho_B} \sum_{i \subset A}^{N_A} \sum_{j \subset B}^{N_B} \frac{\delta(r_{ij} - r)}{4\pi r^2}, \quad \text{(S1)}$$

where ρ_B is the average density of type B atoms around atoms A, N_A and N_B are the number of A and B atoms, respectively, r_{ij} is the distance between two atoms A and B, and δ is the Kronecker delta function.

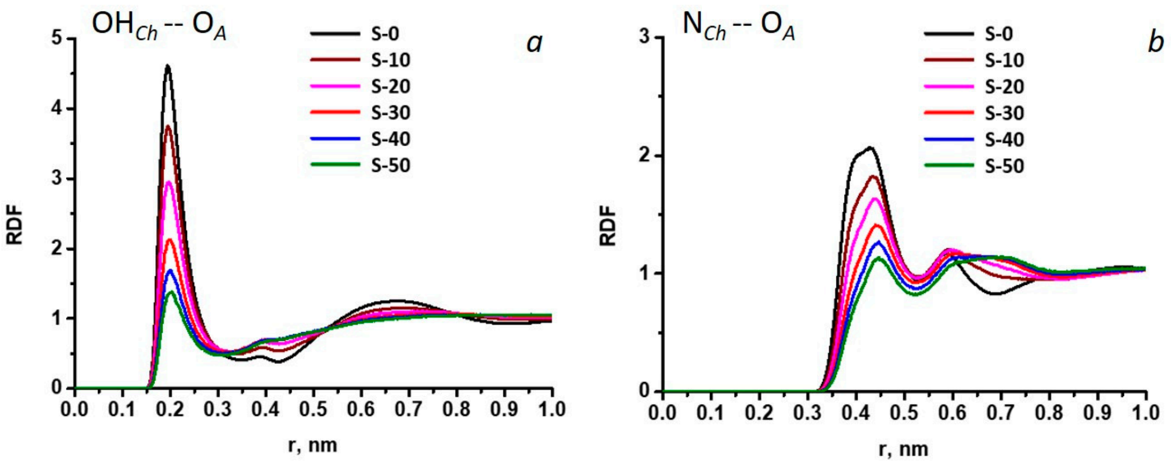

Figure S6. Radial distribution functions for the following pairs: HO_{Ch}-O_A (a), N_{Ch}-O_A (b).

The coordination numbers were calculated as amounts of molecules in the first coordination shells using equation S2.

$$n(r') = 4\pi\rho_B \int_0^{r'} g_{AB}(r) r^2 dr. \quad \text{(S2)}$$

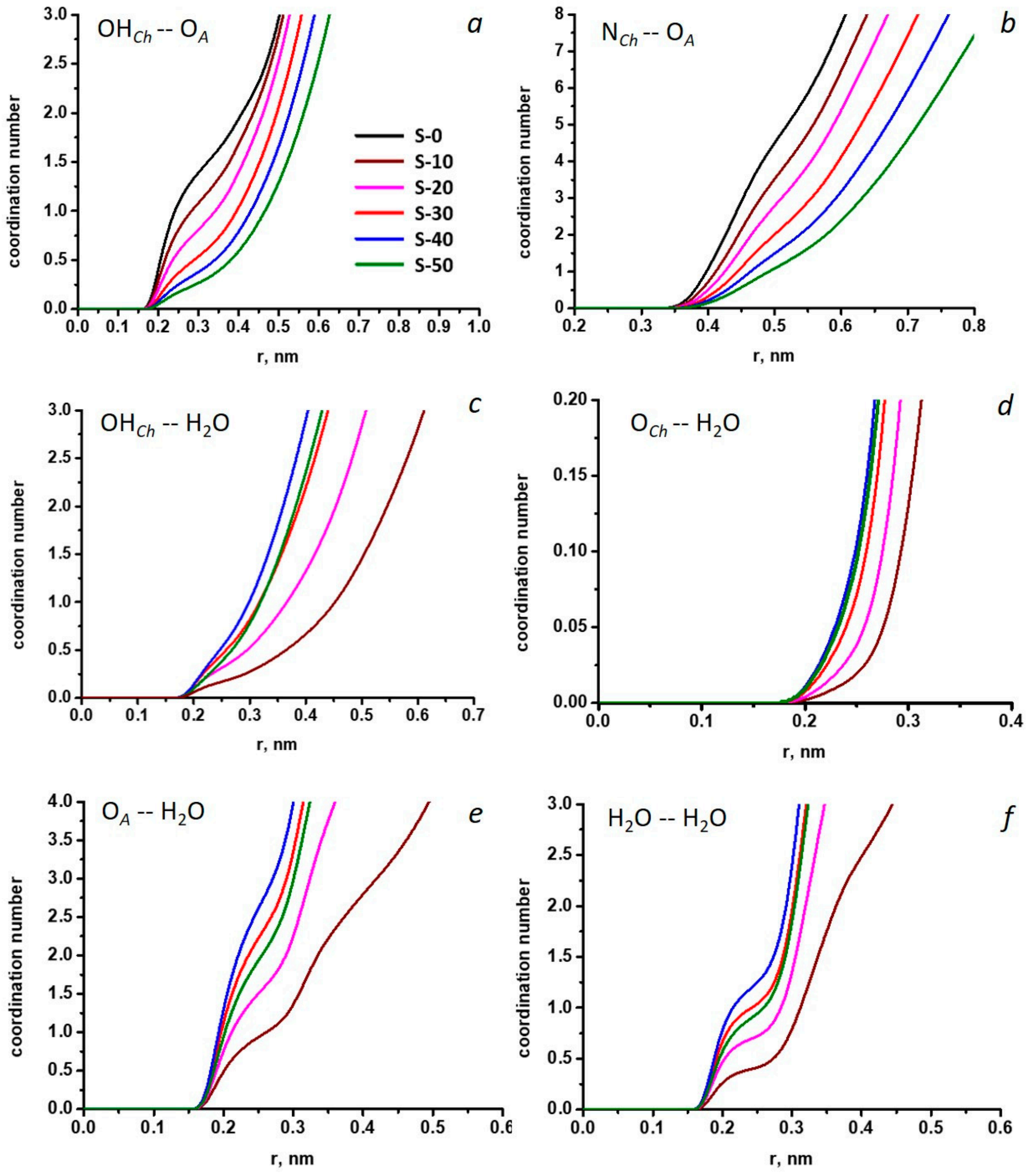

Figure S7. Coordination numbers for the following interactions: HO_{Ch}-O_A (a), N_{Ch}-O_A (b), HO_{Ch}-H_2O (c), O_{Ch}-H_2O (d), O_A-H_2O (e) and H_2O-H_2O (f).

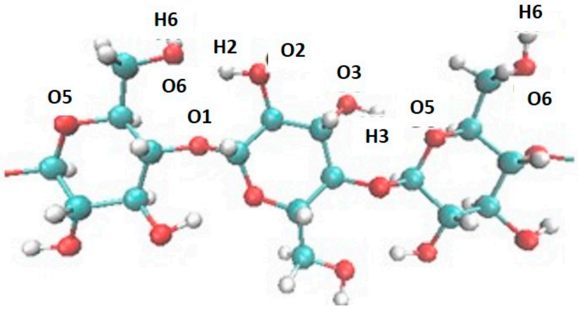

Figure S8. Fragment of the cellulose molecule with numbering the atoms participating in the interactions of the cellulose surface with acrylic anion, choline cation and water atoms.

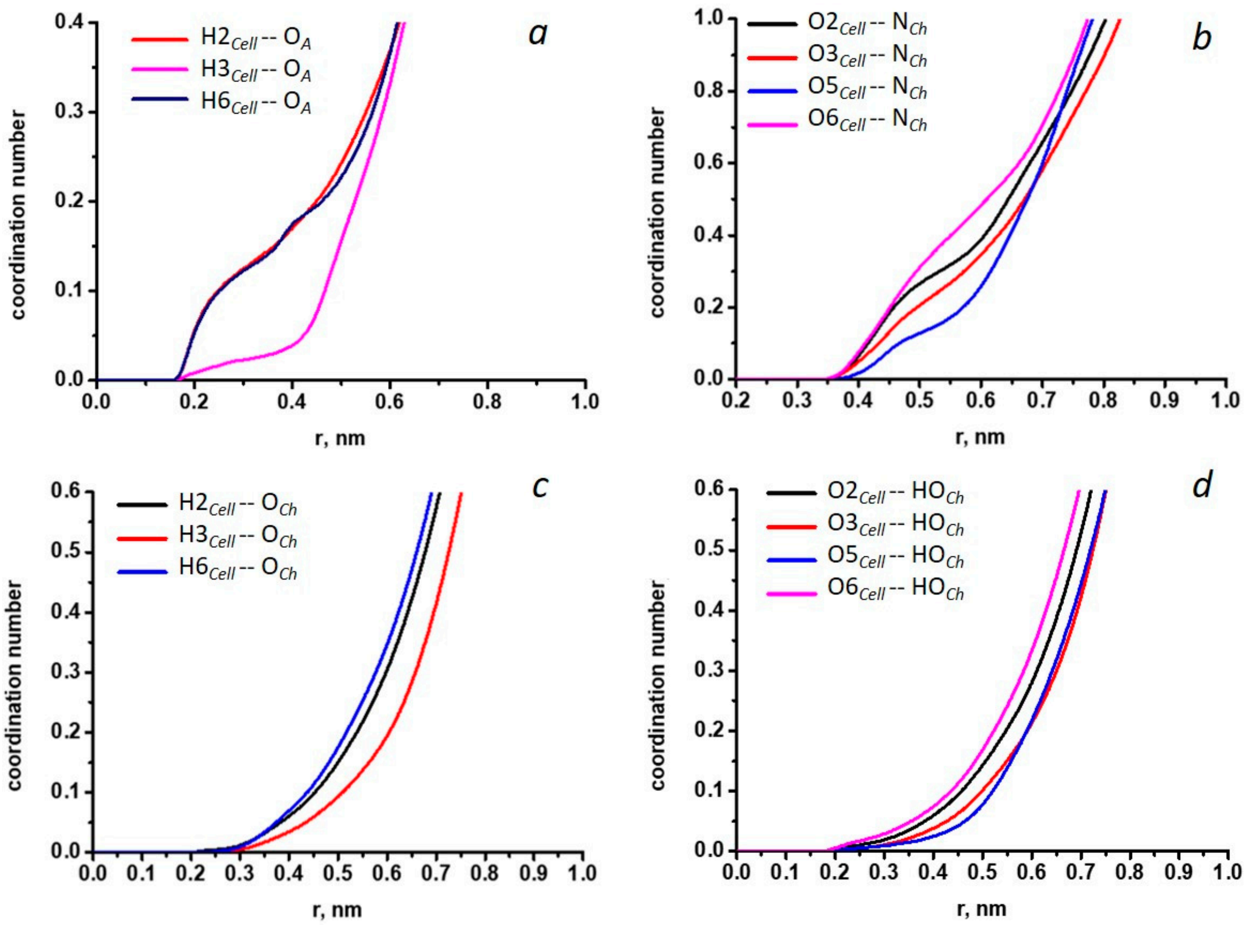

Figure S9. Coordination numbers for hydrogen and oxygen atoms of the primary and secondary hydroxyl groups of cellulose and oxygen atoms of glucose cycle with oxygen atoms of acrylic anions (a), nitrogen atoms of choline cations (b), oxygen atoms of choline hydroxyl groups (c) and hydrogen atoms of choline hydroxyl groups (d) for the system without water. Oxygen atoms (O1) connecting glucose cycles are not achievable for the IL components and the data for them are not presented.

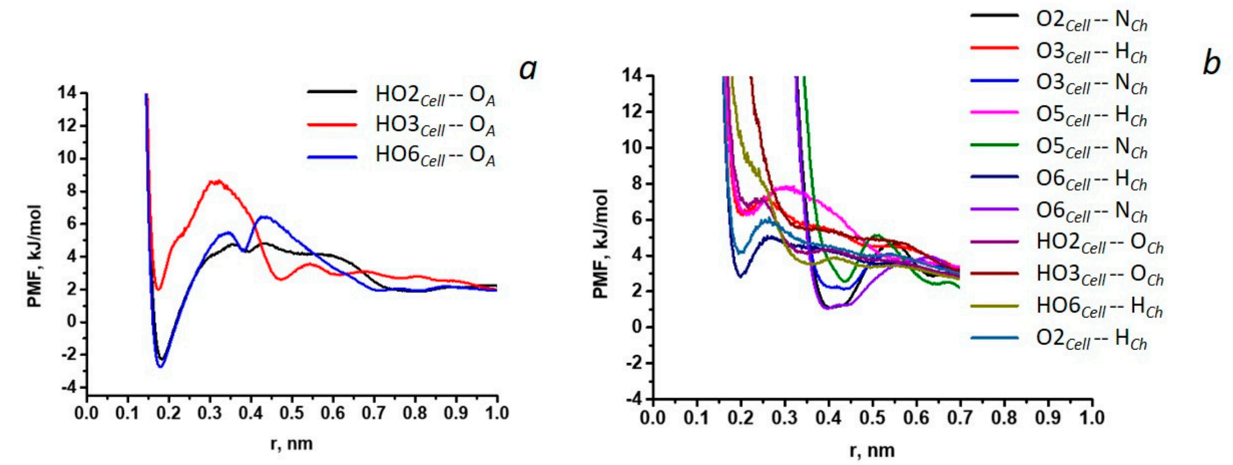

Figure S10. Potentials of mean force for oxygen atoms of the primary and secondary hydroxyl groups of cellulose and oxygen atoms of acrylic anion (a) and for oxygen atoms of the primary and secondary hydroxyl groups and glucose cycles of cellulose with nitrogen atoms of choline cations and with hydrogen atoms of choline hydroxyl groups as well as hydrogen atoms of cellulose hydroxyl groups with oxygen atoms of choline hydroxyl groups (b).

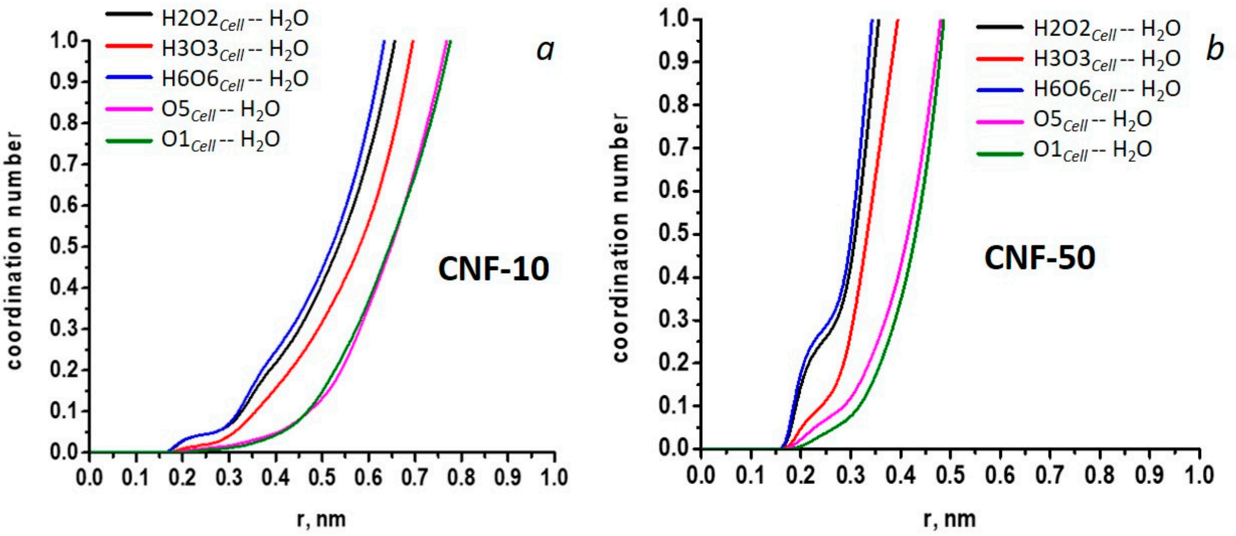

Figure S11. The presented dependencies demonstrate amounts of water contacts with active groups and atoms of cellulose monomers. The coordination numbers for hydrogen and oxygen atoms of the primary and secondary hydroxyl groups of cellulose and oxygen atoms in glucose cycles and bridging glucose cycles with oxygen and hydrogen atoms of water were summed for CNF-10 (a) and CNF-50 (b).

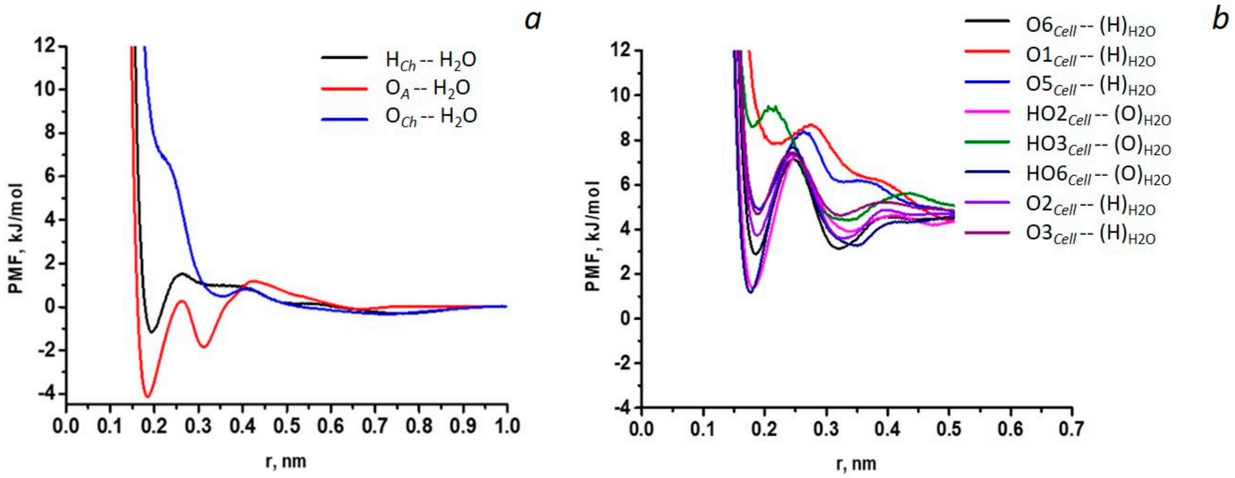

Figure S12. Potentials of mean force for the interactions of choline cation (oxygen and hydrogen atoms) and oxygen atoms of acryl anions with oxygen and hydrogen atoms of water (a) and hydrogen and oxygen atoms of primary and secondary hydroxyl groups of cellulose and oxygen atoms in glucose cycles and bridging glucose cycles with oxygen and hydrogen atoms of water (b).

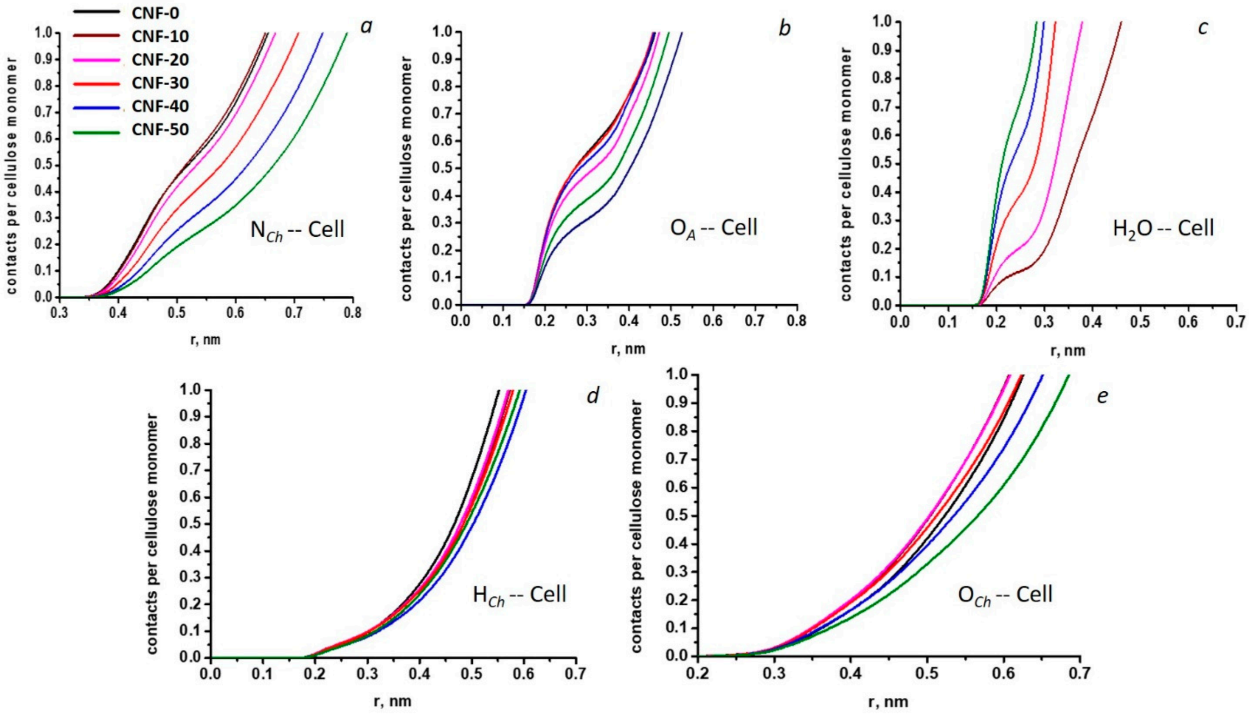

Figure S13. The numbers of the contacts per cellulose monomer were obtained by summing coordination numbers for hydrogen and oxygen atoms of the primary and secondary hydroxyl groups of cellulose and oxygen atoms of glucose cycle with nitrogen atoms of choline cations (a), oxygen atoms of acrylic anions (b), oxygen and hydrogen atoms of water (c), hydrogen atoms of choline hydroxyl groups (d) and oxygen atoms of choline hydroxyl groups (e).
